# Notification of new procedures: an issue for all surgeons

**Published:** 2012-10

**Authors:** Bruce Campbell

**Affiliations:** Chair, Interventional Procedures Advisory Committee; Chair, Medical Technologies Advisory Committee; National Institute for Health and Clinical Excellence, MidCity Place, 71 High Holborn, London WC1V 6NA. www.nice.org.uk

NICE finds out about new procedures which are entering practice through notifications via its website; through personal contact with clinicians; and through opportunistic surveillance. Mostly, procedures are notified by clinicians and manufacturers, but managers, commissioners, private insurers and patients sometimes also notify procedures which are of interest or concern to them. I write every 1-2 years to the leaders of all professional medical organisations asking them whether new procedures are emerging in their area of work. Since 2002 there have been 1,105 notifications and these have resulted in publication of 428 pieces of Interventional Procedures guidance (66 of these were reviews).

The numbers of notifications have fluctuated during the last 10 years with a recent trend to slightly lower numbers. It is unclear whether this is simply one of the fluctuations; whether fewer new procedures are being introduced; or whether there has been any decrease in awareness of the need to notify new procedures to NICE.

The one mandate for notifying new procedures rests on clinicians. Shortly after the Interventional Procedures Programme started a Health Service Circular was published which stipulated that: ‘Medical practitioners intending to carry out a new interventional procedure should seek the approval of their NHS Trust’s Clinical Governance Committee. If the procedure is not listed on NICE’s website (www.nice.org.uk/ip), the Chair of the Committee should notify the procedure to NICE via the website.’ 1 Notification is done via http://www.nice.org.uk/ipsearch/index.jsp. The meaning of a ‘new procedure’ is one that a fully trained clinician has not done before (excluding, of course, a procedure which is well established and which he/she happened not to undertake during training).

There is no bar to clinicians notifying NICE themselves and telling their hospital that they have done so: that is common practice. The onus to inform the hospital concerned and to notify NICE if the procedure is not already on the NICE website applies equally to introducing new procedures in the private sector. That principle was established through agreements between NICE and organisations representing both private hospitals and private insurers.

Fewer than half of all notifications result in a decision for NICE to evaluate the procedure and publish guidance, based on advice from clinical specialists and deliberations which can be complex. For example, procedures may have already been notified, but with a different name, or they may be considered minor variations of established procedures. A fundamental consideration is whether the notified procedure might possibly differ in any aspect of safety or efficacy from related procedures which are established practice.

Interventional procedures guidance aims to protect patients from harm, while facilitating their access to new, beneficial procedures in the best possible circumstances. It offers reassurance to clinicians and to their hospitals that they are introducing procedures in a way which would withstand critical scrutiny. It specifies uncertainties which need to be clarified by further research and /or collection of data. Identifying procedures depends on clinicians notifying them when they first contemplate their use. That does not mean that they cannot start using the procedure: until NICE publishes guidance, the kind of ‘special arrangements’ recommended when evidence is uncertain is the rule: tell your hospital; tell your patients; and audit your results with special care.
